# A bone-based 3D scaffold as an *in-vitro* model of microenvironment–DLBCL lymphoma cell interaction

**DOI:** 10.3389/fonc.2022.947823

**Published:** 2022-10-18

**Authors:** Jessica Ceccato, Maria Piazza, Marco Pizzi, Sabrina Manni, Francesco Piazza, Ilaria Caputo, Francesco Cinetto, Lorena Pisoni, Diletta Trojan, Riccardo Scarpa, Renato Zambello, Angelo Paolo Dei Tos, Livio Trentin, Gianpietro Semenzato, Fabrizio Vianello

**Affiliations:** ^1^ Hematology Unit, Department of Medicine, University of Padua, Padua, Italy; ^2^ Laboratory of Myeloma and Lymphoma Pathobiology, Veneto Institute of Molecular Medicine (VIMM) and Foundation for Advanced Biomedical Research (FABR), Padua, Italy; ^3^ Surgical Pathology and Cytopathology Unit, Department of Medicine-DIMED, University of Padua, Padua, Italy; ^4^ Internal Medicine and Allergology and Clinical Immunology Units, Treviso Ca’ Foncello Hospital, Treviso, Italy; ^5^ Treviso Tissue Bank Foundation, Treviso, Italy

**Keywords:** diffuse B-cell lymphoma, scaffold, mesenchymal stroma cell, extracellular matrix, 3D, organoid

## Abstract

About 30% of patients with diffuse large B-cell lymphoma (DLBCL) relapse or exhibit refractory disease (r/r DLBCL) after first-line immunochemotherapy. Bone marrow (BM) involvement confers a dismal prognosis at diagnosis, likely due to the interaction between neoplastic cells and a complex tumor microenvironment (TME). Therefore, we developed a 3D *in-vitro* model from human decellularized femoral bone fragments aiming to study the role of mesenchymal stromal cells (MSC) and the extracellular matrix (ECM) in the adaptation, growth, and drug resistance of DLBCL lymphoma cells. The 3D spatial configuration of the model was studied by histological analysis and confocal and multiphoton microscopy which allowed the 3D digital reproduction of the structure. We proved that MSC adapt and expand in the 3D scaffold generating niches in which also other cell types may grow. DLBCL cell lines adhered and grew in the 3D scaffold, both in the presence and absence of MSC, suggesting an active ECM–lymphocyte interaction. We found that the germinal center B-cell (GCB)-derived OCI-LY18 cells were more resistant to doxorubicin-induced apoptosis when growing in the decellularized 3D bone scaffold compared to 2D cultures (49.9% +/- 7.7% Annexin V^+^ cells in 2D condition compared to 30.7% + 9.2% Annexin V^+^ 3D adherent cells in the ECM model), thus suggesting a protective role of ECM. The coexistence of MSC in the 3D scaffold did not significantly affect doxorubicin-induced apoptosis of adherent OCI-LY18 cells (27.6% +/- 7.3% Annexin V^+^ 3D adherent cells in the ECM/MSC model after doxorubicin treatment). On the contrary, ECM did not protect the activated B-cell (ABC)-derived NU-DUL-1 lymphoma cell line from doxorubicin-induced apoptosis but protection was observed when MSC were growing in the bone scaffold (40.6% +/- 5.7% *vs*. 62.1% +/- 5.3% Annexin V^+^ 3D adherent cells *vs*. 2D condition). These data suggest that the interaction of lymphoma cells with the microenvironment may differ according to the DLBCL subtype and that 2D systems may fail to uncover this behavior. The 3D model we proposed may be improved with other cell types or translated to the study of other pathologies with the final goal to provide a tool for patient-specific treatment development.

## Introduction

Diffuse large B-cell lymphoma (DLBCL) is the most common non-Hodgkin lymphoma (NHL) worldwide and accounts for about 40% of new NHL cases annually (1). Over the last decade, the treatments of lymphoma patients have enormously grown, thanks to the development of new drugs and drug combinations. Nevertheless, around 30% of patients relapse or exhibit refractory disease (r/r DLBCL) (2). The presence of bone marrow (BM) involvement has been consistently identified among negative prognostic factors (3). In this regard, the interaction of tumor cells with the tumor microenvironment (TME) is a key determinant of intratumor heterogeneity in DLBCL, potentially affecting prognostically relevant features (4, 5).

BM mesenchymal stromal cells (BM-MSC) are known regulators of cellular proliferation and tissue differentiation. To the same extent, MSC have both anti-inflammatory and immunosuppressive properties, characteristics exploited by tumor cells to escape immune surveillance. Therefore, previous studies demonstrated that MSC had a dual effect on DLBCL, promoting neoplastic cell progression and drug resistance (5).

In a biological context, neoplastic cells are in contact not just with MSC and other cells but also with the extracellular matrix (ECM), a macromolecule network that comprehends proteins, glycoproteins, and proteoglycans. ECM plays a central role in the maintenance of the structural and functional integrity of organs. For instance, in solid tumors, ECM has been shown to facilitate the creation of a tumorigenic microenvironment by promoting angiogenesis and inflammation (6). Nevertheless, the impact of ECM in the biology of NHL is almost unknown even if cluster of differentiation 44 (CD44), a complex transmembrane glycoprotein, has been shown to potentially play a role in lymphoid tumor growth in the BM (7).

Conventional approaches evaluating the complex interaction of the microenvironment with lymphoma cells are based on two-dimensional (2D) systems. These 2D systems represent oversimplified tools, which are not sufficiently adequate to mimic the tumor microenvironment. To overcome this issue, the development of three-dimensional (3D) *in-vitro* models has taken hold in recent years to allow a deeper understanding of cell–cell and cell–ECM interactions (8).

Several different 3D techniques have been developed in diverse research fields, each with its own advantages and limitations (9). Existing 3D systems in the study of lymphoma are scanty and based on spheroids which, although effective for the study of complex structures involving multiple cell types, do not mimic the tissue architecture (10, 11). Therefore, we developed a 3D *in-vitro* model, based on the human bone scaffold, which preserves the native biochemical and biophysical characteristics of the BM compartment, combined with human primary MSC cells, with the aim of proposing a tool to deeply explore the DLBCL microenvironment.

## Methods

### Scaffold preparation

**Figure 1:**
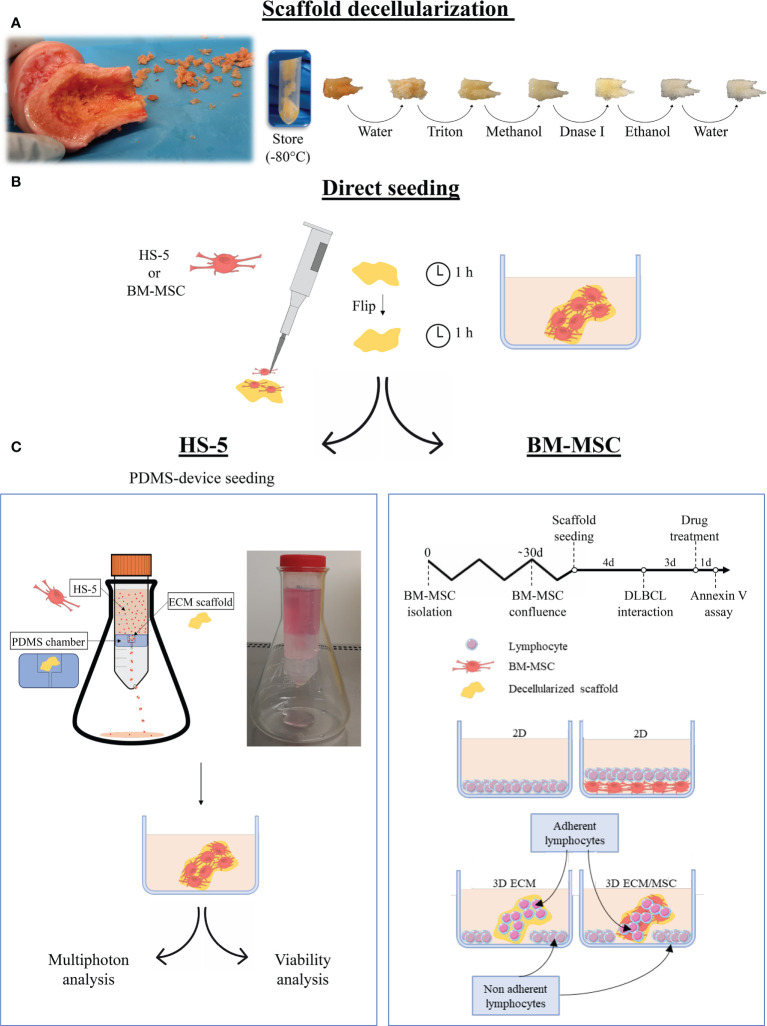
Schematization of the main phases of the study. **(A)** Following fragmentation of human femoral bones, fragments were decellularized with complete removal of cellular components. **(B)** The fragments were then recellularized with HS-5 or bone marrow mesenchymal stromal cells (BM-MSC) by direct seeding, placing half of the amount of cells on each side of the fragment. The cell culture medium was added after an additional incubation hour. The following steps depended on the cells used for the recellularization: **(C)** left panel: HS-5 recellularized fragments underwent polydimethylsiloxane (PDMS) device seeding (double-step seeding procedure) to improve and streamline the recellularization. The PDMS device consisted of a PDMS chamber containing the bone scaffold, placed in a 50-ml Falcon tube holed at the base. The HS-5 cell suspension flowed by gravity through the PDMS chamber, allowing the cell to adhere to the scaffold. The models realized through the HS-5 double-step seeding procedure were used for multiphoton analysis and viability assays. **(C)** Right panel: the BM-MSC recellularized model did not undergo double seeding by the PDMS device as one step recellularization was effective after 4 days of culture. DLBCL was then added and fragments were incubated for additional 3 days before drug treatment. Annexin V assay was performed after 24 h of treatment. For drug treatments, the following conditions were evaluated: a conventional 2D lymphocyte culture, a 2D lymphocyte/BM-MSC co-culture, a 3D culture of neoplastic lymphocytes only, and a 3D lymphocyte/BM-MSC co-culture. In the 3D systems, both adherent and non-adherent lymphocytes were analyzed. Pictures were partly created by smart.servier.com.

Human femoral bones (kindly provided by Fondazione Banca dei Tessuti, Treviso, Italy) were cut into axial sections approximately 2 mm thick. The bone was modeled to a final geometry of 2 mm × 2 mm × 1 mm (length × depth × thickness). The entire bone and the fragments were stored at −80°C (**Figure 1A**).

To remove cellular material, thawed fragments were rinsed overnight in filtered Milli-Q water at 4°C, placed 48 h in sterile 1% Triton X-100 (Euroclone, Milano, Italy) in phosphate-buffered saline (PBS without calcium and magnesium; Euroclone, Italy) and then in absolute methanol for 24 h. To remove all residual DNA, the fragments were treated with DNase I 0.1 mg/ml (Sigma-Aldrich, Italy) at 37°C for 2 h. The fragments were then washed in PBS by continuous gradual shaking, treated with absolute ethanol for 4 h, and rinsed with filtered Milli-Q water for 2 h. All steps were performed in constant agitation. The decellularized fragments were then stored at −80°C.

### Cell cultures and 3D model development

All cell lines, primary cells, and 3D models were cultured at 37°C with 5% CO_2_. HS-5 adherent stromal cells (ATCC, American Type Culture Collection) were cultured in T25 ventilated flasks (Falcon) in Dulbecco’s modified Eagle’s medium [DMEM; high glucose with L-glutamine, without sodium pyruvate (Euroclone, Italy) with 1% penicillin/streptomycin (P/S) (Euroclone, Italy) and 10% fetal bovine serum (FBS; Euroclone, Italy)].

To analyze the 3D structure of the model using a multiphoton microscope, HS-5 mCherry^+^ cells were used. For cell transduction, 30,000 cells/well in a 48-well plate were infected with mCherry lentiviral particles containing the EX-NEG-Lv216 plasmid (GeneCopoeia, Rockville, MD, USA) and purified using a third-generation lentiviral packaging system. The transduction was carried out in a DMEM cell culture medium containing 8 µg/ml of polybrene (Sigma-Aldrich, Italy) at 37°C overnight. After 24 h, a complete DMEM cell culture medium without polybrene was replaced. An mCherry^+^ stable HS-5 cell clone was obtained through puromycin selection (0.5 µg/ml) that was initiated 2 days after transduction. A titration curve for puromycin resistance was previously obtained by an antibiotic kill curve assay (data not shown).

DLBCL cell lines, OCI-LY18 and NU-DUL-1 (DSMZ, Deutsche Sammlung von Mikroorganismen und Zellkulturen, Germany), were cultured in T25 ventilated flasks in RPMI 1640 (Euroclone, Italy) cell culture medium with 1% P/S, 0.05 mM of 2-β-mercaptoethanol, 10% FBS, and 20% FBS, respectively.

For BM-MSC isolation, 3 ml of BM aspirate was obtained from subjects who underwent staging procedures for NHL lymphomas. Only samples that did not show lymphoma infiltration were considered for MSC cultures. All samples were obtained with written informed consent in accordance with local ethical committee requirements. BM aspirates were treated with a lysis buffer for at least 10 min to lyse erythrocytes and centrifuged at 1,000 rpm for 3 min. Then, the pellet was resuspended and gently washed in 0.9% sodium chloride solution; centrifuged again at 1,000 rpm for 3 min; resuspended in 5 ml of DMEM with 20% FBS, 1% P/S, 0.01 mg/ml of fungizone, and 0.25 μg/ml of ciprofloxacin; and plated in T25 flasks (Costar, Cambridge, MA, USA). When 90%–95% confluence was raised (after about 30 days), adherent cells were trypsinized (Gibco, UK) and expanded for 3–5 weeks. BM-MSC were checked for positivity of CD105, CD73, and CD90 and for the lack of expression of CD45 and CD20 (data not shown).

The 3D models were cultured in 12-well plates with 1.5 ml of cell culture medium. Before seeding, frozen fragments were thawed and rinsed in the cell culture medium for about 30 min.

For MSC recellularization of the scaffold, 0.5 × 10^6^ HS-5 cells were directly seeded on a decellularized bone fragment with a micropipette. Half of the cells were resuspended in 10 μl of cell culture medium and seeded on one side of the fragment. After 1 h, the fragments were turned on the other side and seeded with the remaining cell suspension.

As shown in **Figure 1C**, according to the type of cell used, recellularized models followed a different path: BM-MSC recellularized models were directly used for the experiment, whereas an additional recellularization was recommended for HS-5 recellularized fragments. The double seeding was performed by a polydimethylsiloxane (PDMS, Sylgard 184) device. The PDMS device was developed by placing a silicone chamber inside a 50-ml Falcon tube. The PDMS chamber, realized through a mold, consisted of a compartment containing the bone fragment and a conduit (**Figure 1C**, left panel). The cell suspension (4.5 × 10^6^ HS-5 suspended in 25 ml of cell culture medium) was allowed to flow through the chamber at a flow rate of 9.4 ml per minute. The device was held by an Erlenmeyer flask and incubated at 37°C with 5% CO_2_ in a traditional cell incubator. The PDMS device seeding was performed on previously directly seeded fragments, after 2 days of incubation.

Primary cell recellularization of the scaffold was performed by directly seeding 6 × 10^4^ BM-MSC. Due to the dimension of primary MSC and the difficulty to obtain them, the double-step procedure was performed only for HS-5 cells. Recellularized scaffolds were then placed into a 12-well plate with 1.5 ml of cell culture medium.

Lymphoma cell seeding on scaffolds was performed as follows: 0.75 × 10^6^ OCI-LY18 or NU-DUL-1 was resuspended in 1.5 ml of cell culture medium and cultured with the scaffold in a 12-well plate. Some lymphocytes autonomously adhered to the scaffold generating a 3D culture; the others precipitate to the bottom of the well without getting in touch with the scaffold realizing a usual 2D culture (**Figure 1C**, right panel). When 2D and 3D co-cultures were performed, the lymphocyte cell culture medium was used.

### DNA extraction

Fragments were incubated at 56°C in a thermomixer for 48 h with 500 μl of mix constituted by 250 μl lysis buffer (urea 8 M; EDTA 20 mM pH 8; SDS 1%; Tris–HCl 0.2 M pH 8; NaCl 0.4 M), 25 μl SDS 10% (Amresco, Solon, OH, USA), 12.5 μl Proteinase K (PanReac Applichem, Darmstadt, Germany, 20 mg/ml), and water to the volume. After 13,000 rpm 4°C centrifugation, the supernatant was transferred into 1 ml of frozen ethanol and centrifuged again at 13,000 rpm for 30 min at 4°C (Thermo Fisher Scientific, Waltham, MA, USA, Heraeus Fresco 17 centrifuge). The DNA pellet was air-dried and resuspended in water.

### Cytofluorometric assays

Cells were harvested from the model by a 3-min trypsin treatment, resuspended in a cell culture medium, and washed with PBS before Annexin V/propidium iodide (PI) assay (Immunostep, Barcelona, Spain) or staining with PE anti-CD19 (Becton Dickinson, Franklin Lakes, New Jersey, USA), FITC anti-CD45 (BD, 345808), FITC anti-CD20 (BD, 345792), and PE anti-CD105 (Caltag Laboratories, Carlsbad, USA, MHCD10504) conjugated antibodies, according to the manufacturers’ recommendations.

A FACS Canto II (BD) cytometer was used and 10,000 events/sample were analyzed using the FACS DIVA Software v8 0.2.

### Drug treatment

The ECM/MSC model was prepared by seeding 6 × 10^4^ BM-MSC as described above. The same number of cells was seeded in a 12-well plate to perform the 2D co-culture. BM-MSC were allowed to grow for 4 days. Then, 0.75 × 10^6^ OCI-LY18 or NU-DUL-1 was added with 1.5 ml of cell culture medium.

After 72 h, the time required for DLBCL cell adhesion and stabilization in the scaffold, 0.7 or 1 μM of doxorubicin (Selleckem, USA) was added, respectively, to NU-DUL-1 and OCI-LY18 for an additional 24 h. The testing concentration was selected after a dose–response investigation: the percentage of apoptosis was evaluated 24 h after doxorubicin administration at concentrations of 0.001, 0.01, 0.1, 0.3, 0.5, 0.7, 1, and 10 μM (data not shown). Lymphocytes were harvested from the models after 3 min of trypsin treatment and analyzed with Annexin V cytofluorometric assay. In the 3D conditions, we considered both adherent and non-adherent cells. The experimental setting is summarized in **Figure 1C**, right panel.

### 3D model staining and digital reconstruction

After PBS washing, the 3D models were fixed in 4% formalin (15 min) and permeabilized with PBST (0.1% Triton X-100 in PBS) if necessary (15 min).

3D digital reconstruction of the entire structure of the model was performed by exploiting scaffold autofluorescence and HS-5 mCherry^+^ cells. The Multiphoton Galvo System (Scientifica, UK) was used. The average dimension of the reconstructions is 500 × 500 × 100 μm (height, width, depth), and around 400 stacks were acquired for each sample.

The BM-MSC recellularized model was visualized through a confocal microscope (Zeiss LSM900) upon Alexa Fluor 594 Phalloidin staining (A12381, Molecular Probe, USA): stock solution 200 units/ml diluted 1:40 in 1% BSA in PBS and staining at room temperature for 20 min in the dark. Three PBST gentle washing steps were performed after phalloidin staining. Adherent lymphocytes were visualized with the same instrument after staining: anti-CD19 primary antibody (Cell Signaling Technology, USA, 3574S) overnight at 4°C in gentle agitation and secondary Alexa Fluor^®^ 647-conjugated antibody (Abcam, ab150083) for 1 h in the dark at room temperature in gentle agitation. The staining with DAPI 0.001 mg/ml in PBS was performed for 10 min in the dark. By using a confocal microscope, about 50 stacks were acquired and 50-μm-deep reconstructions were performed.

All the staining procedures were performed in 2 ml Eppendorf tubes ensuring that 3D fragments were fully submerged. Therefore, fragments were resumed by forceps and gently and quickly dried with a paper towel. For image acquisitions, fragments were anchored in a 6-well plate using transparent nail polish, normally used in imaging procedures. The wells were filled with deionized water and samples visualized by a ×40 water immersion objective.

Images were analyzed with a Zeiss ZEN 3.2 blue edition program and 3D reconstruction was performed by the 3D viewer plugin of Fiji.

### Histological analysis

Formalin-fixed samples were embedded in paraffin, following standardized protocols obtained from routine diagnostic practice. From each sample, serial 4-μm-thick sections were stained with hematoxylin and eosin and submitted for immunohistochemical analysis. The latter was performed in the BOND-MAX automated immunostainer (Leica Biosystems, Wetzlar, Germany) using the anti-CD20 primary antibody (clone L26, dilution 1:50; Dako, Agilent, Santa Clara, USA). Histological sections were jointly examined by two expert hematopathologists who were blinded to the experimental conditions. Morphometric analyses were performed manually, using the Leica DFC295 digital color camera and LAS X software (Leica Microsystems, Wetzlar, Germany).

## Results

### Scaffold decellularization

**Figure 2:**
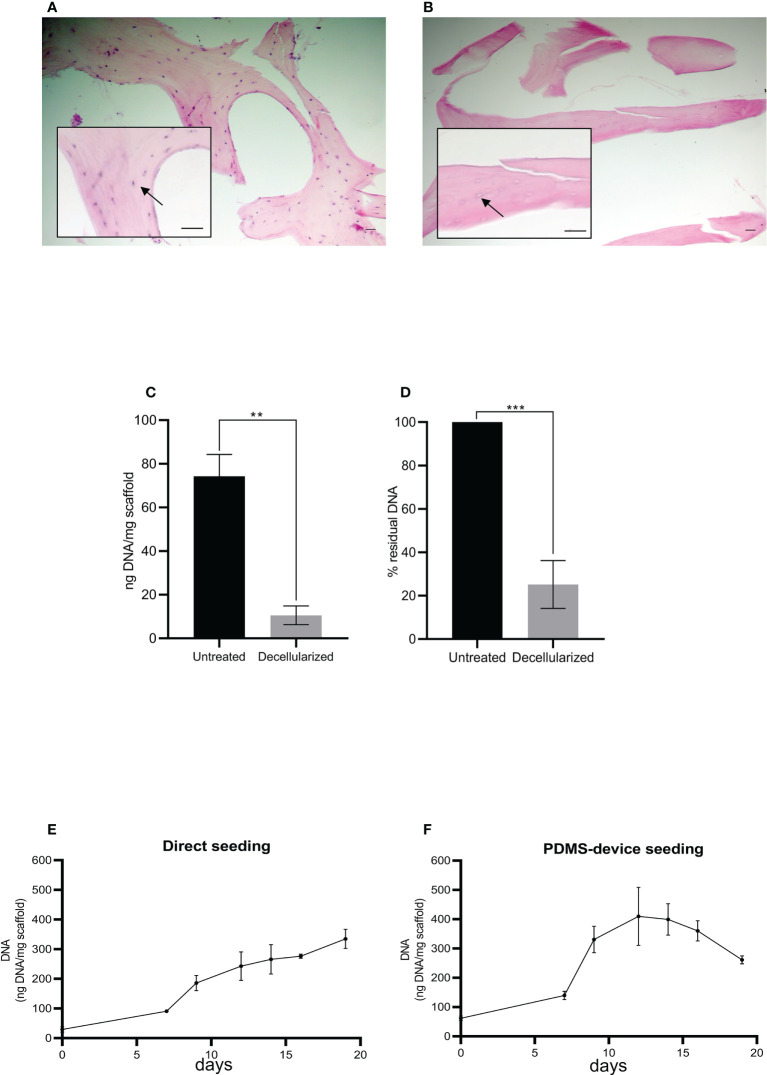
Histological sections of human femoral bone fragment before **(A)** and after **(B)** the decellularization process. Arrows indicate a bone cell **(A)** and the absence of a bone cell **(B)**. Hematoxylin/eosin staining. Scale bar: 100 μm. Histograms show total DNA content in the bone fragments after decellularization compared to the untreated ones **(C, D)**. Statistical analysis performed with unpaired *t*-test; *n* = 3; **p < 0.005; ***p < 0.0005. **(E, F)** The total DNA content (ng), normalized to the weight in mg of the scaffold, as an index of cellular presence. The scaffolds were seeded with HS-5 cells directly **(E)** or directly plus the PDMS device **(F)**. Both seeding techniques allowed the attachment and the expansion of the cells into the scaffold: direct seeding allowed a constant and linear increase of cellular content, while double seeding permitted a quicker and higher recellularization of the scaffold.

The effectiveness of the decellularization protocol (**Figure 1A**) was confirmed by histological analysis and DNA quantitation. As shown in **Figure 2B**, no cells were present after decellularization treatment compared to non-treated fragments (**Figure 2A**). At the same time, the treated fragments showed 10.6 + 6.1 ng of DNA per mg of scaffold compared to 74.3 + 14.2 ng DNA/mg of scaffold found in non-treated fragments (**Figure 2C**), corresponding to a residual DNA content of 15.6% + 13.7% after decellularization (**Figure 2D**). Thus, the decellularization protocol was effective in removing all cellular components as reported in the literature (12).

### Scaffold recellularization: 3D model implementation

#### MSC recellularization

As schematized in **Figure 1B**, decellularized fragments were recellularized with HS-5 or BM-MSC. The progression of recellularization was evaluated by DNA measurements, and data demonstrated that seeding techniques allowed MSC adhesion and expansion in the decellularized scaffold. Although the direct seeding was effective in reaching the complete recellularization of the scaffold, it needed a longer time than the double-step seeding procedure (**Figures 2E, F**). The maximum DNA content reached in direct seeded fragments was 334.8 + 55.8 ng per mg of fragment weight after 20 days of incubation (**Figure 2E**), which was shortened to 12 days by the double seeding method: 409.6 + 171.5 ng of DNA per mg of fragment weight after 12 days of incubation (**Figure 2F**). Indeed, in the double seeding technique, the first step allowed the formation of the cell groundwork, facilitating the attachment of HS-5 cells flowing through the PDMS device. Therefore, the double-step seeding procedure streamlined the MSC recellularization of the scaffold.

We also found a reduction of DNA content on day 20 in reference to day 15 (**Figure 2F**) probably because cells died and detached from the scaffold when the maximum confluence was reached.

**Figure 3:**
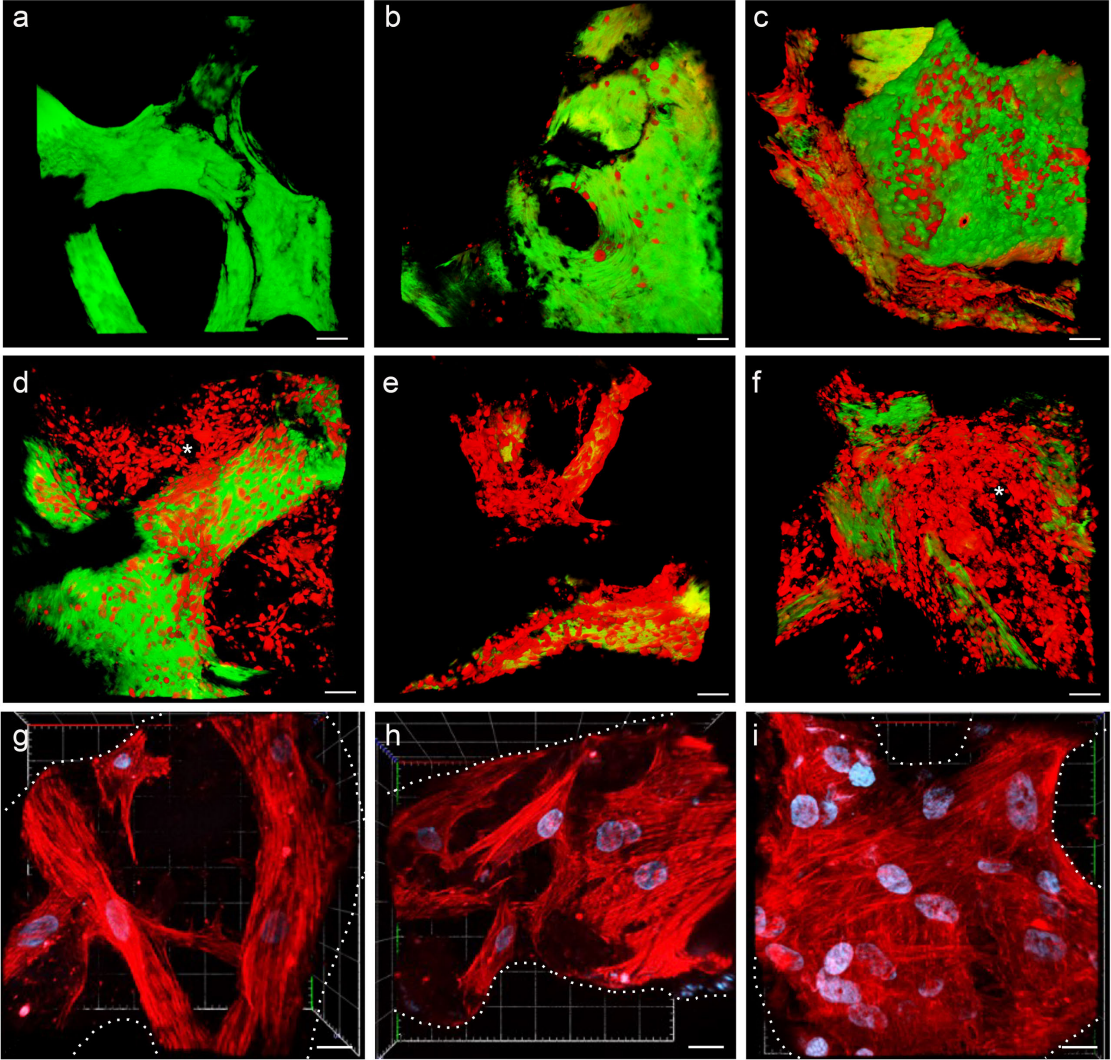
**(A–F)** Multiphoton microscopy 3D reconstruction of HS-5 mCherry^+^ cells grown in the scaffold. **(A)** Decellularized scaffold (autofluorescence). **(B)** 24 h after direct seeding. **(C)** 24 h after PDMS device seeding. The following images represent the model after 8 **(D)**, 14 **(E)**, and 19 **(F)** days of incubation. * indicated MSC forming cellular niches filling the trabecular bone area. Scale bar: 50 μm. Images acquired with the Multiphoton Galvo System (Scientifica, UK) and analyzed with the ImageJ software. **(G–I)** Confocal microscopy 3D reconstruction of BM-MSC grown in the scaffold. Images acquired with a Zeiss confocal microscope after 7 **(G)**, 12 **(H)**, and 15 **(I)** days of incubation and analyzed with the Zeiss ZEN 3.2 blue edition program, and 3D reconstruction was performed by the 3D viewer plugin of Fiji. Blue: DAPI; red: phalloidin. Scale bar: 20 μm.

MSC adhesion and expansion in the 3D scaffold were also demonstrated by multiphoton and confocal microscopy 3D reconstruction performed over time (**Figure 3**). The 3D structure of the scaffold was acquired, thanks to the biological autofluorescence of the ECM scaffold (**Figure 3A**). Then, the model was observed 24 h after HS-5 mCherry^+^ direct seeding (**Figure 3B**), 24 h after PDMS device seeding (**Figure 3C**), and after 8, 14, and 19 days of incubation (**Figures 3D–F**). These 3D digital reconstructions clearly showed the increasing cell content and the spatial distribution of HS-5 in the scaffold. Moreover, images proved the formation of cellular niches in the scaffold (**Figures 3D, F** **)**. Also, BM-MSC showed adhesion and expansion capability in the 3D context (**Figures 3G–I**).

**Figure 4:**
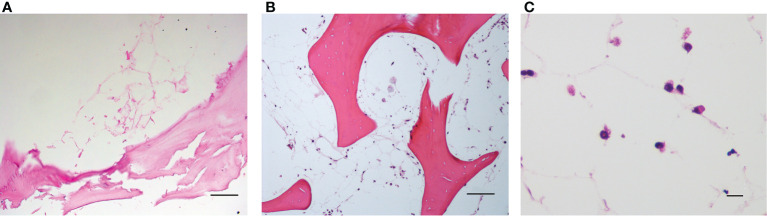
**(A)** Decellularized scaffold showing the extracellular matrix web; **(B, C)** recellularized scaffold after 12 days of incubation, in which HS-5 cells are clearly visible along the matrix web. Structure remaining after adipocyte removal is clearly visible. Hematoxylin/eosin staining. Scale bar: 100 μm **(A, B)**; 10 μm **(C)**.

The spatial distribution of MSC into the scaffold was also analyzed by histological analysis (**Figures 4A–C**). As already reported in **Figure 2B**, **Figure 4A** demonstrates that the 3D extracellular matrix of the trabecular bone and the extracellular structure of adipocytes were preserved after the decellularization process. This extracellular structure facilitated cell adhesion and the formation of cellular niches in the 3D scaffold (**Figures 4B, C**). The distribution pattern of MSC recapitulated the normal pattern of human bone marrow, where abundant reticular cells locate among hematopoietic cells and around adipocytes (13).

HS-5 viability was evaluated by measuring apoptosis after cell recovery from the scaffold. Although we found a trend of decreased apoptosis over time (**Supplementary Figure 1F**), differences were not statistically significant (18.5% + 13.5% of Annexin V^+^ cells after 2 days of incubation, 16.6% + 5.4% after 7 days, and 13.7% + 7.9% at day 9; **Supplementary Figure 1F**). Moreover, HS-5 cells recovered from the scaffold were able to readapt and expand in a conventional culture flask, demonstrating that the entire process from seeding, expansion in the 3D scaffold, and then to harvesting did not affect the growing features of these cells (**Supplementary Figures 1A–D**).

On the contrary, BM-MSC were resistant to trypsin treatment and could not be recovered from the scaffold (**Supplementary Figure 2** and **Supplementary Methods**). Therefore, when 3D ECM/MSC models were treated with trypsin, only lymphocytes were recovered.

#### Lymphocyte interaction

**Figure 5:**
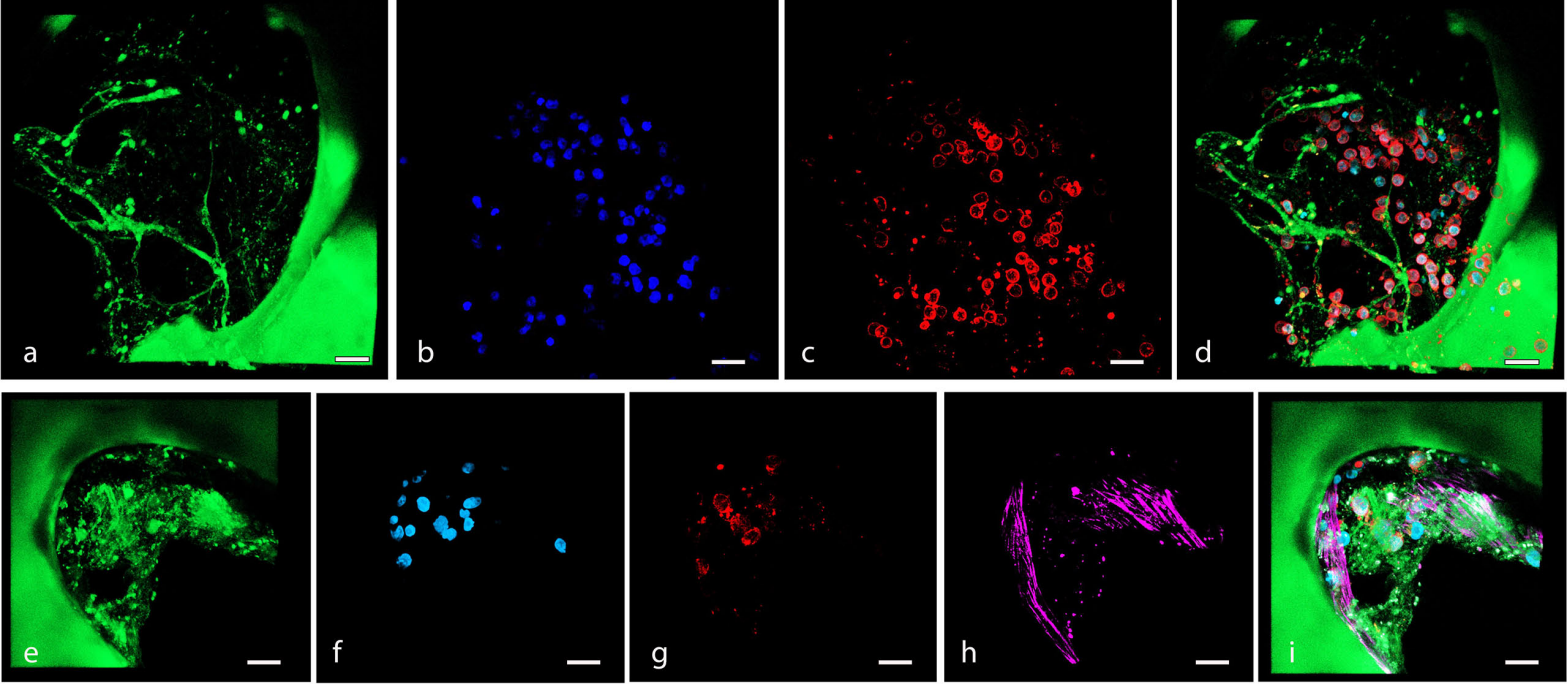
**(A–D)** Confocal microscopy 3D reconstruction of OCI-LY18 that adhered to the scaffold (ECM model): **(A)** scaffold autofluorescence, **(B)** nuclei (DAPI), **(C)** OCI-LY18 (CD19), and **(D)** merge. **(E–I)** Confocal microscopy 3D reconstruction of OCI-LY18 that adhered to the BM-MSC recellularized scaffold (ECM/MSC model): **(E)** scaffold autofluorescence, **(F)** nuclei (DAPI), **(G)** OCI-LY18 (CD19), **(H)** BM-MSC (phalloidin), and **(I)** merge. Scale bar: 100 μm.

**Figure 6:**
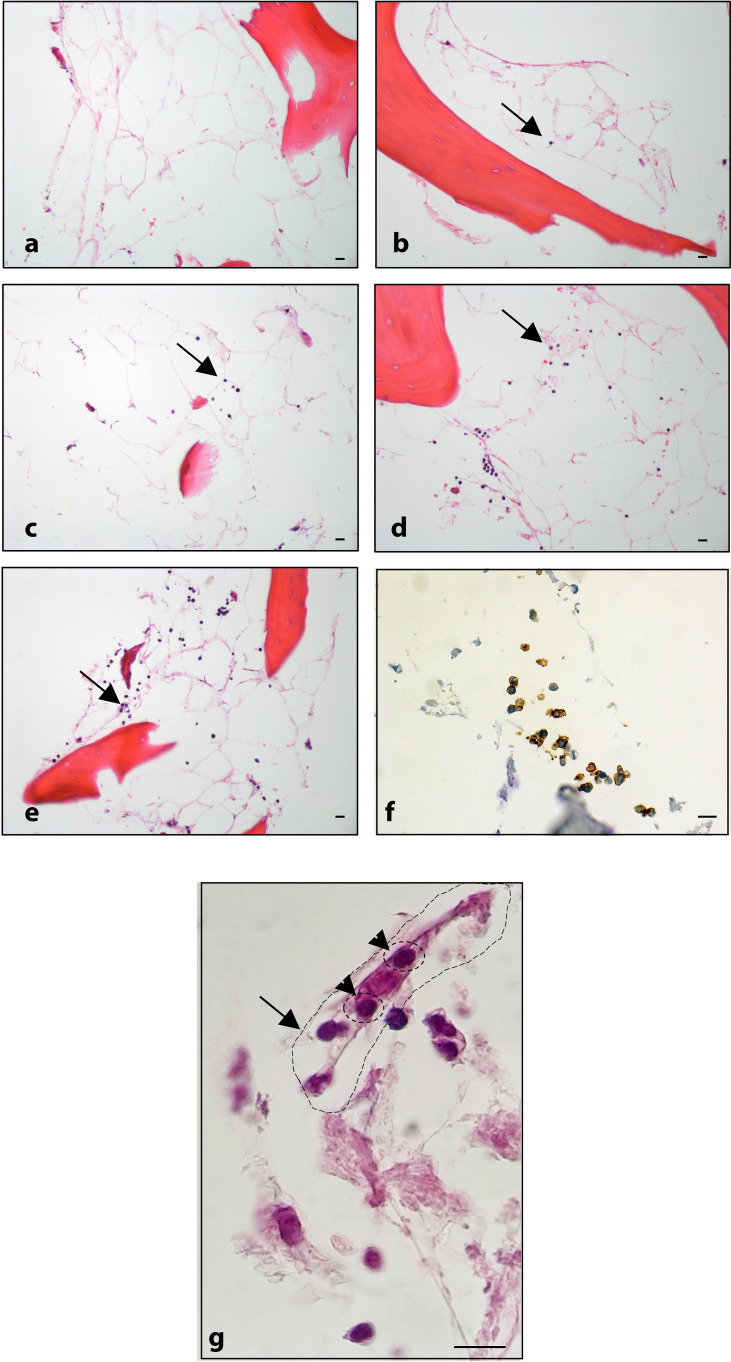
Histological analysis: **(A)** BM-MSC recellularized scaffold; ECM/MSC model after 1, 3, 6, and 8 days of incubation with OCI-LY18 (**B–E**, respectively). **(F)** CD20 staining of OCI-LY18 adherent cells. **(G)** ECM/MSC model: arrow indicates a BM-MSC; head arrows indicate adherent neoplastic cells. Hematoxylin/eosin staining. Scale bars: 10 μm.

The lymphoma cells were autonomously able to migrate, adhere, and expand both in the decellularized (ECM model, **Figures 5A–D**) and BM-MSC recellularized (ECM/MSC model, **Figures 5E–I**) scaffolds as demonstrated by *in-situ* 3D immunofluorescence analysis (**Figure 5**). Histological analysis performed after BM-MSC and DLBCL cell recellularization of the scaffold (ECM/MSC model) showed that neoplastic lymphocytes were able to expand over time (**Figures 6A–F**), creating a strong contact with BM-MSC (**Figure 6G**).

Following adhesion and expansion in the scaffold, lymphocytes harvested from the model maintained the expression of CD19, CD20, and CD45, demonstrating that the seeding and harvesting procedures did not affect the cell surface’s characteristics (**Supplementary Figure 3**).

**Figure 7:**
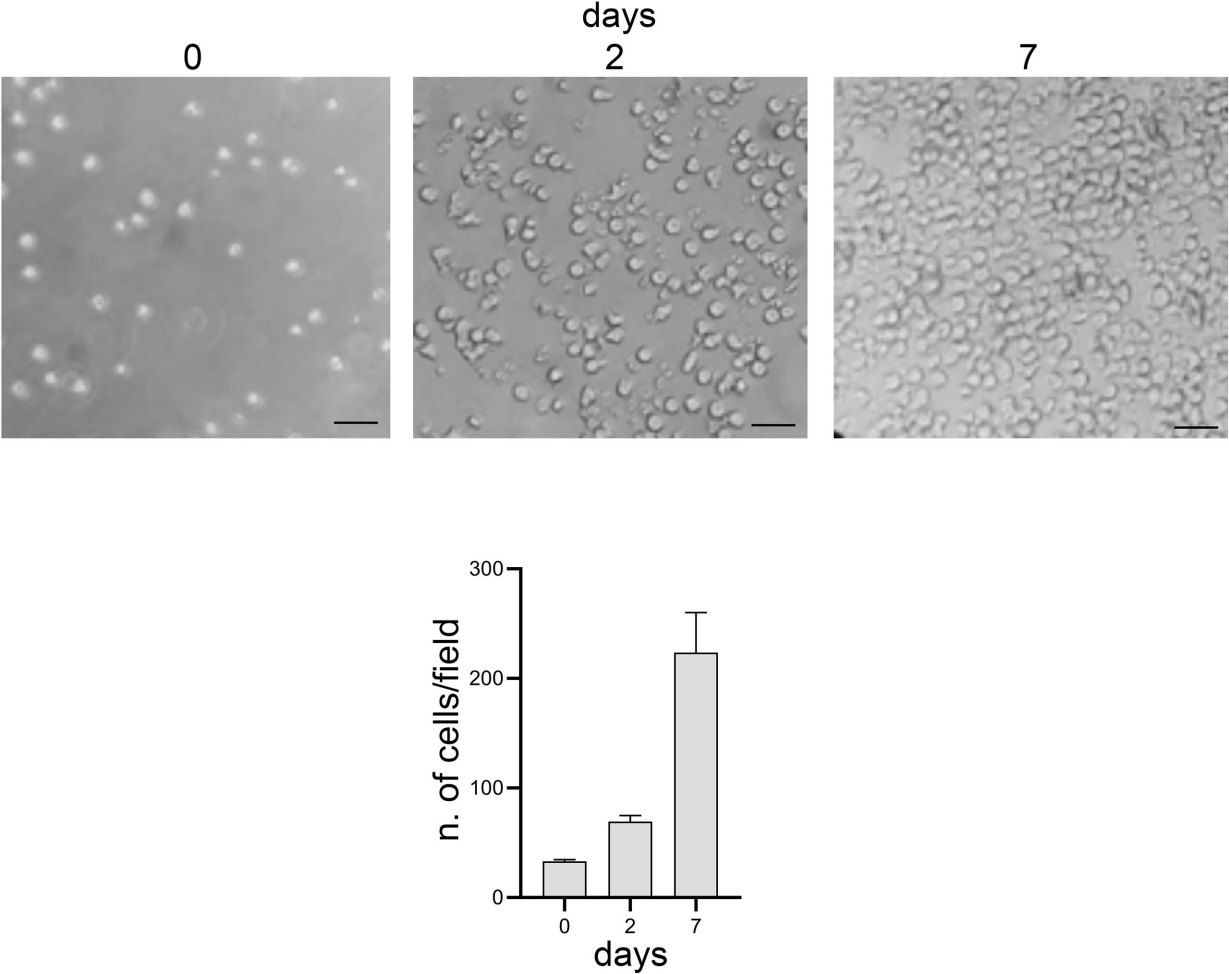
Optical microscope images, bright field. 2D culture of lymphocytes harvested from the 3D model. Scale bar: 20 μm. Graphs represent the average number of cells present per image: 3 images per time point and 6 areas per image were analyzed.

Moreover, lymphocytes harvested from the 3D model were able to readapt and grow in the conventional 2D culture (**Figure 7**).

Interestingly, cytospin-realized microscope slides of lymphocytes harvested from the 3D model (**Supplementary Figure 4** and **Supplementary Methods**) showed the presence of lymphocytes with a pseudopod-like shape, suggesting that the interaction between cells and stroma triggers the organization of the cytoskeleton.

**Figure 8:**
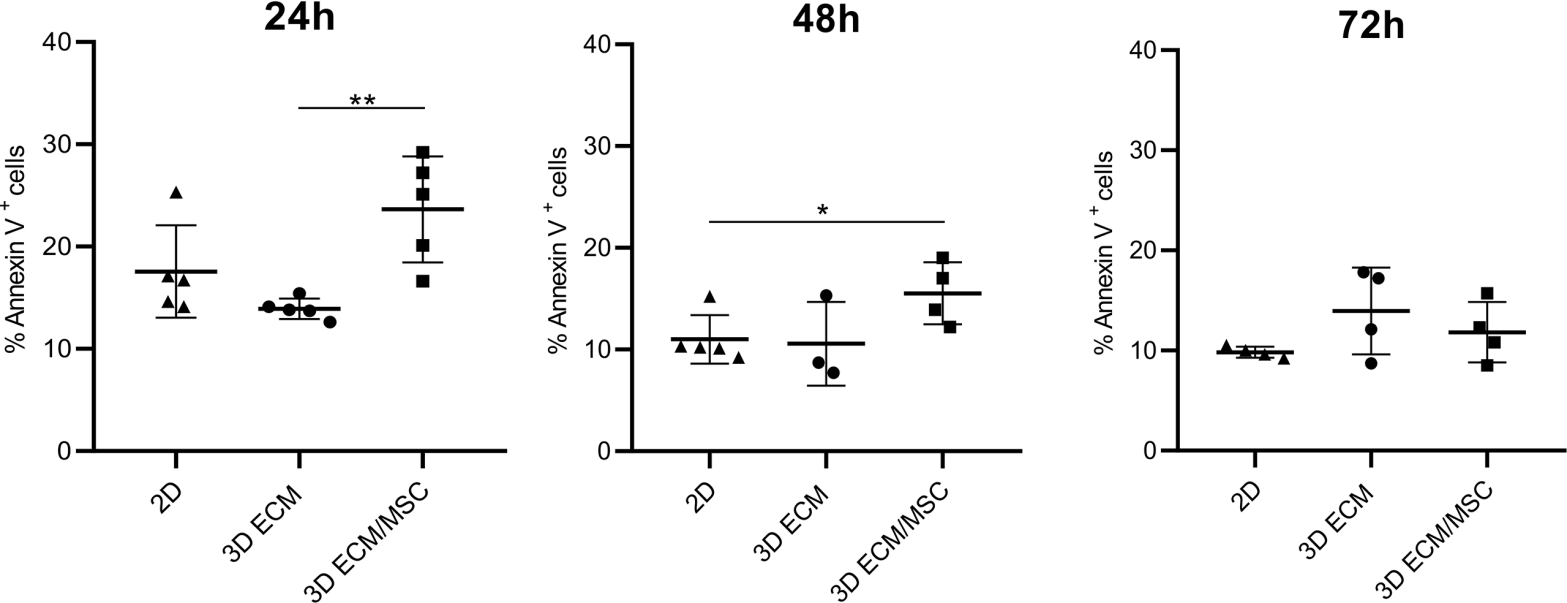
Annexin V/PI cytofluorometric assay of OCI-LY18 cells harvested from the model after 24, 48, and 72 h of incubation. 2D represents a control standard culture; 3D ECM indicates the model constituted by decellularized scaffold and OCI-LY18; 3D ECM/MSC indicates the scaffold recellularized also with primary MSC. The data reported correspond to the sum of Annexin V^+^/PI^−^ and Annexin V^+^/PI^+^ cells. Statistical analysis: non-parametric *t*-test. *p < 0.05; **p < 0.005.

The viability of lymphocytes adapted to the 3D models was quantified by Annexin V/PI cytofluorometric assay. As shown in **Figure 8**, after 24 h of 3D culture, the spontaneous apoptosis level was statistically higher (*p* < 0.005) in lymphocytes co-cultured with BM-MSC (ECM/MSC model: 23.6% + 5.2%) compared to lymphocytes in the absence of BM-MSC (ECM model: 13.9% + 1.1%). Spontaneous apoptosis level progressively reduced after 48 h and became comparable among the different conditions after 72 h (2D: 9.8% + 0.5%; 3D ECM: 13.9% + 4.3%; 3D ECM/MSC: 11.8% + 3.1%; **Figure 8**). These data demonstrated that 72 h of incubation were necessary for 3D adherent lymphocytes to reach a basal apoptotic level comparable to standard culture, suggesting the timing in which the experiments were performed.

Therefore, to investigate whether the 3D microenvironment affected the sensitivity of lymphocytes to antiproliferative compounds, the 3D models were exposed to doxorubicin, a cytotoxic anthracycline antibiotic conventionally used in combination with other chemotherapeutic agents in the treatment of DLBCL, and both adherent and non-adherent lymphocytes were analyzed (the scheme of the experiment is reported in **Figure 1C**, right panel).

**Figure 9:**
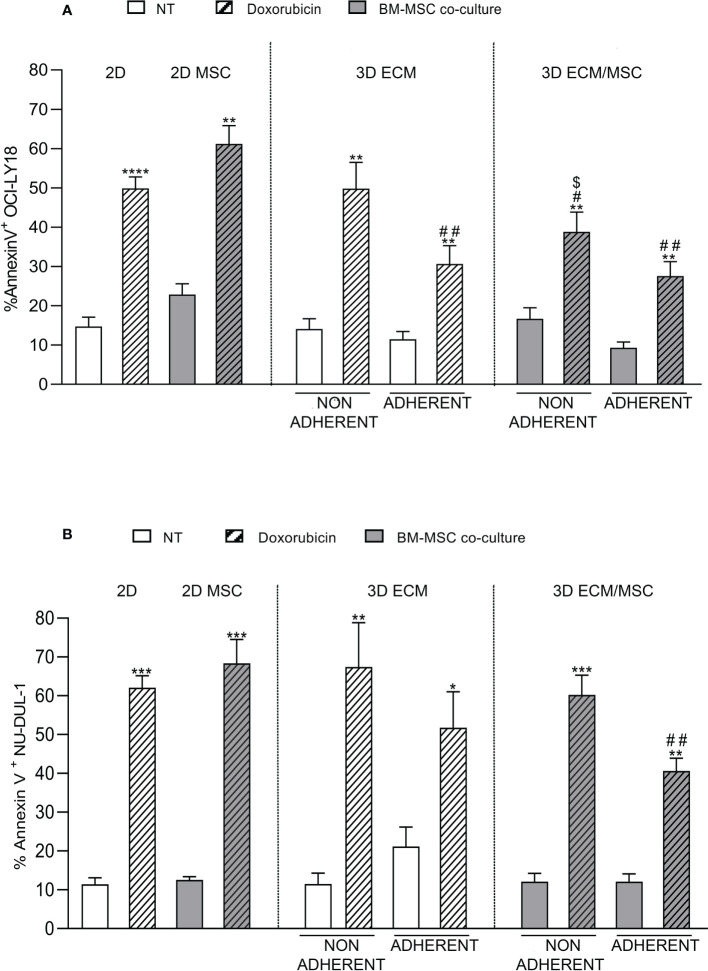
**(A)** Annexin V cytofluorometric assay of OCI-LY18 after doxorubicin treatment. The experimental conditions are summarized in **Figure 1C**, right panel. The apoptotic effect of doxorubicin was reduced in the ECM/MSC model adherent lymphocytes in both cell lines. With the OCI-LY18 cell line, an apoptosis reduction was also found in the ECM model adherent lymphocytes and in non-adherent cells of the ECM/MSC system. Statistical analysis: non-parametric *t*-test. * in relation to the non-treated conditions: *p = 0.05; **p < 0.005; ***p < 0.0001. # in relation to the 2D-treated condition: ^#^ *p* < 0.05; ^##^ *p* < 0.005. $ in relation to the 2D co-culture treated condition: $*p* < 0.05. **(A)** OCI-LY18: *n* = 4; **(B)** NU-DUL-1: *n* = 3.

As reported in **Figure 9A**, the results showed that doxorubicin induced apoptosis in 49.9% + 7.7% of germinal center B-cell (GCB)-derived OCI-LY18 cells in a conventional 2D culture, compared to a 14.7% + 6.3% baseline apoptosis (*p* < 0.0001). Non-adherent cells growing in the ECM 3D model showed a similar level of apoptosis compared to the 2D culture (non-treated: 14.1% + 5.2%; doxorubicin-treated: 49.8% + 13.4%). In the ECM 3D model, doxorubicin-treated adherent OCI-LY18 cells showed a reduced apoptosis level compared to the 2D culture (doxorubicin-treated: 30.7% + 9.2% in the 3D ECM model compared to 49.9% + 7.7% in 2D; *p* < 0.005; **Figure 9A**), indicating a protective role of ECM in doxorubicin-induced apoptosis.

In the ECM/MSC model, the level of doxorubicin-induced apoptosis of adherent OCY-LY18 was still significantly reduced compared to the 2D culture (27.6% + 7.3% in the 3D ECM/MSC model compared to 49.9% + 7.7% in 2D; *p* < 0.005; **Figure 9A**). However, no additive effects were observed compared to apoptosis levels measured in the ECM model (**Figure 9A**).

Moreover, a significant resistance of non-adherent OCI-LY18 cells to doxorubicin-induced apoptosis was also observed in the ECM/MSC 3D model, suggesting that humoral factors released by MSC grown in the 3D model may exert a protective effect (38.9% + 9.9% in non-adherent ECM/MSC condition compared to 49.9% + 7.7% in 2D and 61.2% + 8.1% in 2D co-culture; *p* < 0.05; **Figure 9A**). Interestingly, in 2D co-cultures, we could not observe a significant protective effect of MSC in doxorubicin-induced apoptosis (non-treated: 22.8% + 4.7%; doxorubicin-treated: 61.2% + 8.1%; *p* < 0.005). Doxorubicin-induced apoptosis was then quantified using NU-DUL-1 cells, an activated B-cell (ABC)-derived cell line (**Figure 9B**). In the same conditions, we observed a significant reduction in doxorubicin-induced apoptosis only in the lymphoma cells adherent to the 3D ECM/MSC model compared to the 2D model (40.6% + 5.7% in ECM/MSC adherent conditions compared to 62.1 + 5.3% in the 2D-treated condition; *p* < 0.005). No significant protection from apoptosis was observed in the ECM 3D model compared to cells treated in the 2D system (**Figure 9B**).

## Discussion

Bone marrow involvement occurs in 10%–20% of cases of DLBCL, and it is characterized by aggressive course and poor response to standard polychemotherapy (3). The interaction of tumor cells with and within the microenvironment certainly does play a role in the pathogenesis of this dismal condition. The behavior of tumor cells in the bone marrow is particularly difficult to simulate *in-vitro* as a multitude of cellular and non-cellular components are involved. More importantly, normal and tumor cells are surrounded by 3D essential physical scaffolding called the ECM.

Traditional 2D cell cultures, although informative when exploited to study cell–cell interaction, cannot reproduce the bone marrow 3D architecture in which the cellular interaction also depends on a complex 3D ECM network.

Not only the spatial architecture of 3D models significantly differs from 2D systems but also cell phenotype, activation, motility, and gene expression are profoundly different (14). Moreover, the response of cancer cells to chemotherapy has been demonstrated to differ when cells are grown in 3D models compared to their 2D monolayer counterparts.

In this paper, we proposed a 3D model suitable for studying the behavior and response to treatments of lymphoma cells adapted to a bone scaffold. Bone fragments were easily and consistently generated from tissue bank-derived bone tissue. Moreover, they were preserved and exploited as an off-the-shelf scaffold for recellularization.

The generation of a bone marrow microenvironment could also be realized by engineered techniques where cells, ECM, and biophysical signals can be controlled *in-vitro*. As an example, a 3D hydrogel can be exploited to mimic the interaction of endothelial and stromal cells in cancer studies (15, 16). Unfortunately, these 3D scaffolds cannot entirely recapitulate our 3D model as they fail to mimic the stiffness and biochemical composition of native bone niche.

In fact, a synthetic 3D model emulating the bone, thus incorporating minerals and collagen as an anchor for cells, is yet to be proposed. Certainly, among the new strategies, the 3D-printed and bioprinted models can provide novel perspectives for reproducing the composition and architecture of bone which may be exploited in cancer studies. However, as the development of 3D models in this field is still at an early stage, our model based on native bone scaffold may help to provide insights into the complexity of the bone microenvironment (17–19).

We demonstrated that an MSC cell line and primary MSC seeded into the scaffold were able to adapt and expand, keeping their biological properties and high viability over time.

Recellularization can be optimized by a double-step protocol based on a PDMS device which generates a slow-flowing cell suspension into the scaffold, significantly speeding up the recellularization by a slow-growing cell type, in our case an MSC cell line.

By 3D digital reconstruction and histological analysis, we demonstrated the generation of MSC niches into the scaffold, mainly located in the net ECM structure left from adipocyte removal. The physical and biological properties of the MSC cell line were maintained for up to 9 days of culture in cells recovered from the scaffold, demonstrating the efficacy of the seeding, culturing, and harvesting procedures.

We found that lymphoma cell lines seeded into the scaffold are selected to autonomously adhere to the bone, independently from the presence of MSC, then progressively growing and keeping their original phenotype and the ability to readapt and expand in a canonical 2D culture. Interestingly, we observed shape changes with pseudopodia-like formation in DLBCL cells, which may point out to active cell–ECM interaction and lymphoma cell cytoskeleton reorganization triggered by ECM. It is tempting to speculate that recellularization of the bone scaffold may select lymphoma cells with autonomous capability to adhere and grow in the bone microenvironment, thus providing a source of cells with specific biological characteristics, distinct from non-adherent cells. This may explain the fact that we did not observe complete recellularization of the scaffold by lymphocytes. Certainly, alternative seeding techniques, for example, recellularization under perfused conditions, may better recapitulate how tumor cells approach and adapt to the bone marrow microenvironment. ECM is crucial for tissue homeostasis and normal organ development. At the same time, the remodeling of the ECM can promote solid tumor progression through mechanisms modulating cell proliferation and apoptosis (20). Therefore, cell adhesion receptors may play a role in lymphoma dissemination. There is evidence that adhesion receptors not only regulate the trafficking of lymphocytes but they can also mediate the tissue-specific dissemination of lymphomas. Indeed, several adhesion receptors, through the interactions with their ligands, regulate lymphoma cell trafficking and the interaction with ECM. For instance, cellular adhesion molecules (CAMs), L-selectin, and CD44s have been shown to affect dissemination and overall survival in aggressive NHLs (7, 21–23). Our data and this background support the concept that lymphoma cells are able to strongly interact with ECM and that this interaction may foster their growth in the bone marrow microenvironment.

We also found that the chemosensitivity of lymphoma cells adherent to the bone scaffold depends on the ECM and MSC differently, based on their cell-of-origin subtype. In fact, the chemosensitivity of the GCB-derived cell line OCI-LY18 was impaired by interaction with the ECM, and these cells did not seem to gain protection from the coexistence of MSC. On the contrary, protection from doxorubicin was observed in ABC-derived NU-DUL-1 cells only when MSC were growing in the bone scaffold. Of relevance, 2D cultures failed to detect any significant effect of MSC on tumor cell chemosensitivity.

Evidence that adherence to ECM can hamper chemosensitivity of tumor cells has been extensively provided in solid tumors (24). This ground is still substantially unexplored in lymphomas. Rudelius et al. elegantly showed that the proliferation, survival, and migration of mantle cell lymphoma cells were increased by the interaction with integrins and ECM through the expression of focal adhesion kinase (FAK) (25).

The presence of MSC in the tumor microenvironment may translate into different regulatory functions of tumor growth and progression. In fact, MSC can act bidirectionally within the tumor stroma both as cancer-associated tumor-inhibitory MSC and as tumor-supporting MSC (26). These opposite MSC functions depend on the current status of the MSC and the type, threshold, and synergy of local stimuli. Cytokine-dependent “licensing” and the generation of apoptotic MSC have been shown to be required for the generation of the immunomodulatory properties of MSC (3, 27, 28). Therefore, the fact that we did not observe protection from doxorubicin-induced cell death in 2D co-cultures is not entirely unexpected. On the contrary, protection of non-adherent lymphoma cells by MSC in 3D cultures suggests that soluble factors are produced as the results of the interaction of MSC with the bone scaffold, again pointing to an active cell–ECM interaction. Of note, protection of DLBCL from spontaneous and drug-induced apoptosis has been recently shown to rely on soluble factors instead of cell–cell contact (5).

Finally, with regard to the COO-specific interaction of MSC and ECM, our findings add further support to the concept that the tissue microenvironment strongly influences several biological characteristics of tumor cells, from their clonal diversification and intratumor heterogeneity to response to treatment (29). This observation underlines that alternative treatments targeting the microenvironment may have a role in the treatment of DLBCL with bone marrow involvement.

In conclusion, a 3D model of lymphoma adaptation to bone scaffold integrated with MSC interaction may be of help in the evaluation of tumor growth which relies on cell–cell or cell–ECM interactions. Particularly, sensitivity and resistance to a given treatment could be investigated. Ultimately, our model can be exploited to develop a 3D environment repopulated not only by stromal cells but also by other constituents of the immune system, as well as components of the vasculature, with the aim of ultimately getting closer to model patient tumors.

## Data availability statement

The raw data supporting the conclusions of this article will be made available by the authors, without undue reservation.

## Ethics statement

The studies involving human participants were reviewed and approved by the Ethics Committee of the Padova University Hospital internal Institutional Board. The patients/participants provided written informed consent to participate in this study.

## Author contributions

JC and FV conceived and coordinated the study, designed the experiment, carried out the data analysis, and wrote the paper. JC, MPa, MPi, LP, and IC designed and performed the experiments. FC, RS, FP, RZ, LT, DT, AT and SM coordinated the study and made scientific contributions. GS revised the paper. All authors contributed to the article and approved the submitted version.

## Funding

This research was funded by Gilead Sciences s.r.l. Fellowship Program 2019 to FV.

## Acknowledgments

The authors gratefully acknowledge the financial support of Giorgia Libero Onlus.

## Conflict of interest

The authors declare that the research was conducted in the absence of any commercial or financial relationships that could be construed as a potential conflict of interest.

## Publisher’s note

All claims expressed in this article are solely those of the authors and do not necessarily represent those of their affiliated organizations, or those of the publisher, the editors and the reviewers. Any product that may be evaluated in this article, or claim that may be made by its manufacturer, is not guaranteed or endorsed by the publisher.

## References

[1] Siegel RL Miller KD Fuchs HE Jemal A. Cancer statistics, 2022. CA: A Cancer J Clin (2022) 72:7–33. doi: 10.3322/caac.21708 35020204

[2] Rovira J Valera A Colomo L Setoain X Rodríguez S Martínez-Trillos A. Prognosis of patients with diffuse large b cell lymphoma not reaching complete response or relapsing after frontline chemotherapy or immunochemotherapy. Ann Hematol (2015) 94:803–12. doi: 10.1007/s00277-014-2271-1 PMC4374121 25501975

[3] Yao Z Deng L Xu-Monette ZY Manyam GC Jain P Tzankov A. Concordant bone marrow involvement of diffuse large b-cell lymphoma represents a distinct clinical and biological entity in the era of immunotherapy. Leukemia (2018) 32:353–63. doi: 10.1038/leu.2017.222 PMC5985660 28745330

[4] Dumontet E Mancini SJC Tarte K. Bone marrow lymphoid niche adaptation to mature b cell neoplasms. Front Immunol (2021) 12:784691. doi: 10.3389/fimmu.2021.784691 34956214 PMC8694563

[5] Zhong W Zhu Z Xu X Zhang H Xiong H Li Q. Human bone marrow-derived mesenchymal stem cells promote the growth and drug-resistance of diffuse large b-cell lymphoma by secreting IL-6 and elevating IL-17A levels. J Exp Clin Cancer Res (2019) 38:73–89. doi: 10.1186/s13046-019-1081-7 PMC6373150 30755239

[6] Schmitz R Wright GW Huang DW Johnson CA Phelan JD Wang JQ. Genetics and pathogenesis of diffuse Large b-cell lymphoma. N Engl J Med (2018) 378:1396–407. doi: 10.1056/NEJMoa1801445 PMC6010183 29641966

[7] Bartolazzi A Jackson D Bennett K Aruffo A Dickinson R Shields J. Regulation of growth and dissemination of a human lymphoma by CD44 splice variants. J Cell Sci (1995) 108(Pt 4):1723–33. doi: 10.1242/jcs.108.4.1723 7542258

[8] Rodrigues J Heinrich MA Teixeira LM Prakash J. 3D *In vitro* model (R)evolution: Unveiling tumor–stroma interactions. Trends Cancer (2021) 7:249–64. doi: 10.1016/j.trecan.2020.10.009 33218948

[9] Pape J Emberton M Cheema U. 3D cancer models: The need for a complex stroma, compartmentalization and stiffness. Front Bioeng Biotechnol (2021) 9:660502. doi: 10.3389/fbioe.2021.660502 33912551 PMC8072339

[10] Foxall R Narang P Glaysher B Hub E Teal E Coles MC. Developing a 3D b cell lymphoma culture system to model antibody therapy. Front Immunol (2020) 11:605231. doi: 10.3389/fimmu.2020.605231 33628205 PMC7897703

[11] Weiswald L-B Bellet D Dangles-Marie V. Spherical cancer models in tumor biology. Neoplasia (2015) 17:1–15. doi: 10.1016/j.neo.2014.12.004 25622895 PMC4309685

[12] Li M Zhang T Jiang J Mao Y Zhang A Zhao J. ECM coating modification generated by optimized decellularization process improves functional behavior of BMSC. Mater Sci Eng C Mater Biol Appl (2019) 105:110039. doi: 10.1016/j.msec.2019.110039 31546422

[13] Abe-Suzuki S Kurata M Abe S Onishi I Kirimura S Nashimoto M. CXCL12 stromal cells as bone marrow niche for CD34 hematopoietic cells and their association with disease progression in myelodysplastic syndromes. Lab Invest (2014) 94:1212–23. doi: 10.1038/labinvest.2014.110 25199050

[14] Mabry KM Payne SZ Anseth KS. Microarray analyses to quantify advantages of 2D and 3D hydrogel culture systems in maintaining the native valvular interstitial cell phenotype. Biomaterials (2016) 74:31–41. doi: 10.1016/j.biomaterials.2015.09.035 26433490 PMC4661067

[15] Bersini S Jeon JS Dubini G Arrigoni C Chung S Charest JL. A microfluidic 3D *in vitro* model for specificity of breast cancer metastasis to bone. Biomaterials (2014) 35:2454–61. doi: 10.1016/j.biomaterials.2013.11.050 PMC3905838 24388382

[16] Ghajar CM Peinado H Mori H Matei IR Evason KJ Brazier H. The perivascular niche regulates breast tumour dormancy. Nat Cell Biol (2013) 15:807–17. doi: 10.1038/ncb2767 PMC3826912 23728425

[17] Zhang YS Duchamp M Oklu R Ellisen LW Langer R Khademhosseini A. Bioprinting the cancer microenvironment. ACS Biomater Sci Eng (2016) 2:1710–21. doi: 10.1021/acsbiomaterials.6b00246 PMC5328669 28251176

[18] Moroni L Burdick JA Highley C Lee SJ Morimoto Y Takeuchi S. Biofabrication strategies for 3D *in vitro* models and regenerative medicine. Nat Rev Mater (2018) 3:21–37. doi: 10.1038/s41578-018-0006-y 31223488 PMC6586020

[19] Fischetti T Di Pompo G Baldini N Avnet S Graziani G. 3D printing and bioprinting to model bone cancer: The role of materials and nanoscale cues in directing cell behavior. Cancers (2021) 13:4065–87. doi: 10.3390/cancers13164065 PMC8391202 34439218

[20] Pickup MW Mouw JK Weaver VM. The extracellular matrix modulates the hallmarks of cancer. EMBO Rep (2014) 15:1243–53. doi: 10.15252/embr.201439246 PMC4264927 25381661

[21] Jacob MC Agrawal S Chaperot L Giroux C Gressin R Le Marc’Hadour F. Quantification of cellular adhesion molecules on malignant b cells from non-hodgkin’s lymphoma. Leukemia (1999) 13:1428–33. doi: 10.1038/sj.leu.2401517 10482995

[22] Drillenburg P Pals ST. Cell adhesion receptors in lymphoma dissemination. Blood (2000) 95:1900–10. doi: 10.1182/blood.V95.6.1900 10706853

[23] Tzankov A Pehrs A-C Zimpfer A Ascani S Lugli A Pileri S. Prognostic significance of CD44 expression in diffuse large b cell lymphoma of activated and germinal centre b cell-like types: a tissue microarray analysis of 90 cases. J Clin Pathol (2003) 56:747–52. doi: 10.1136/jcp.56.10.747 PMC1770073 14514777

[24] Henke E Nandigama R Ergün S. Extracellular matrix in the tumor microenvironment and its impact on cancer therapy. Front Mol Biosci (2019) 6:160. doi: 10.3389/fmolb.2019.00160 32118030 PMC7025524

[25] Rudelius M Rosenfeldt MT Leich E Rauert-Wunderlich H Solimando AG Beilhack A. Inhibition of focal adhesion kinase overcomes resistance of mantle cell lymphoma to ibrutinib in the bone marrow microenvironment. Haematologica (2018) 103:116–25. doi: 10.3324/haematol.2017.177162 PMC5777199 29079592

[26] Galland S Stamenkovic I. Mesenchymal stromal cells in cancer: a review of their immunomodulatory functions and dual effects on tumor progression. J Pathol (2020) 250:555–72. doi: 10.1002/path.5357 PMC7217065 31608444

[27] Vianello F Villanova F Tisato V Lymperi S Ho K-K Gomes AR. Bone marrow mesenchymal stromal cells non-selectively protect chronic myeloid leukemia cells from imatinib-induced apoptosis *via* the CXCR4/CXCL12 axis. Haematologica (2010) 95:1081. doi: 10.3324/haematol.2009.017178 20179085 PMC2895031

[28] Duijvestein M Wildenberg ME Welling MM Hennink S Molendijk I van Zuylen VL. Pretreatment with interferon-γ enhances the therapeutic activity of mesenchymal stromal cells in animal models of colitis. Stem Cells (2011) 29:1549–58. doi: 10.1002/stem.698 21898680

[29] Sangaletti S Iannelli F Zanardi F Cancila V Portararo P Botti L. Intra-tumour heterogeneity of diffuse large b-cell lymphoma involves the induction of diversified stroma-tumour interfaces. EBioMedicine (2020) 61:103055. doi: 10.1016/j.ebiom.2020.103055 33096480 PMC7581880

