# Supplementary material for: A bone-based 3D scaffold as an in-vitro model of microenvironment–DLBCL lymphoma cell interaction
Source: Front Oncol. 2022 Oct 18;12:947823. doi: 10.3389/fonc.2022.947823 (PMC9623125; doi:10.3389/fonc.2022.947823)
Supplement: Supplementary file 1 [file DataSheet_1.pdf]

SUPPLEMENTARYMETHODS

Cytospin microscope slides

3D models were treated with 100 µl of trypsin (Gibco, UK) for 3 minutes. Then, the activity of trypsin was neutralized with 100 µl of complete medium and the resulting suspension was introduced in a cytospin support according to manufacturer's recommendations. Microscope slides were stained in pure May-Grunwald (Sigma-Aldrich, Italy) for 3 minutes, in a diluted May-Grunwald solution (1:2 in distilled water) for 3 minutes and in a diluted Giemsa (Sigma-Aldrich, Italy) solution (1:100 in distilled water) for 25 minutes. Then, the colored slides were gently washed with distilled water, air dried and observed with an optical microscope.

Staining for imaging

For vimentin staining, MSC were plated on glass coverslips in 24 well plates and cultured to confluence. Then, they were washed in PBS three times, fixed with 4% paraformaldehyde for 10 min and permeabilized with PBS added with 0.3% Triton for 10 minutes at room temperature. Cells were washed again with PBS, blocked with 2% bovine serum albumin (BSA. VWR Lifes Science) at room temperature and incubated overnight at 4 °C with a monoclonal antibody against Vimentin (1:200; Invitrogen, San Giuliano Milanese, Italy). After three PBS washings, cells were incubated with Rhodamine Red-X-conjugated goat anti–mouse IgG secondary antibody (1:400; Jackson ImmunoResearch, West Grove, PA) for 1 hour in the dark. Glass coverslips with cells were mounted using a DAPI-containing mounting medium (Sigma-Aldrich, St. Louis, MO). Immunoreactivity signals were visualized with the Olympus BX60 microscopy.

Supplementary Figure 1.

a-d) A standard culture of HS-5 cells harvested from the 3D model, 24h a) and 7 days b) after harvesting. The cells maintained their morphological characteristics, the capability to duplicate and the ability to reach the confluence after 21 days c). The cells harvested from the 3D model were morphologically identical to those before seeding d). Scale bar: 10 µm. e) Histogram represents the average number of cells present per field (6 fields per image were analyzed; f) Annexin V/PI test of HS-5 cells harvested from the model over time. Data reported corresponds to the sum between the percentage of Annexin V^{+}/ PI^{-} and Annexin V^{+}/ PI^{+} cells.

Supplementary Figure 2.

Images which provided evidence that the 3 minutes trypsin treatment was not able to detach BM-MSC from the scaffold. a-b) Optical microscopy, bright field. Scale bar: 50 µm. a) 2D primary MSC culture before seeding. b) 24h after trypsinized surnatant culture (2D). No cells were present in the plate. c-d) Optical microscopy, vimentin and DAPI fluorescence. Scale bar: 10 µm. c) 2D BM-MSC culture before seeding. d) Cytospin-realized microscope slide with trypsinized surnatant. No cells were present. The dashed line highlights a scaffold residual fragment. e-f) Confocal microscopy 3D reconstructions, phalloidin and DAPI fluorescence. Scale bar: 20 µm. e) 3D reconstruction of BM-MSC adherent to the scaffold before trypsin treatment. f) 3D reconstruction of BM-MSC adherent to the scaffold after trypsin treatment. Trypsinization process was not able to detach primary MSC from the scaffold.

Supplementary Figure 3.

OCI-LY18 cytofluorimetric characterization. Cells maintain the positivity for CD19 (PE), CD20 (FITC) and CD45 (FITC) and the negativity for CD105 (PE) after seeding and harvesting procedures. 10.000 events per sample were analyzed.

Supplementary Figure 4.

Optical microscope images. Cytospin realized microscope slides of OCI-LY18 cells harvested from the 3D ECM scaffold. Giemsa May-Grunwald staining. a) OCI-LY18 regular circular shape; b-d) OCI-LY18 modified shape. Black arrows indicate pseudopode-like structure. Scale bar: 2 μm.
