# Supplementary figures and images for: A bone-based 3D scaffold as an in-vitro model of microenvironment–DLBCL lymphoma cell interaction
Source: Front Oncol. 2022 Oct 18;12:947823. doi: 10.3389/fonc.2022.947823 (PMC9623125; doi:10.3389/fonc.2022.947823)

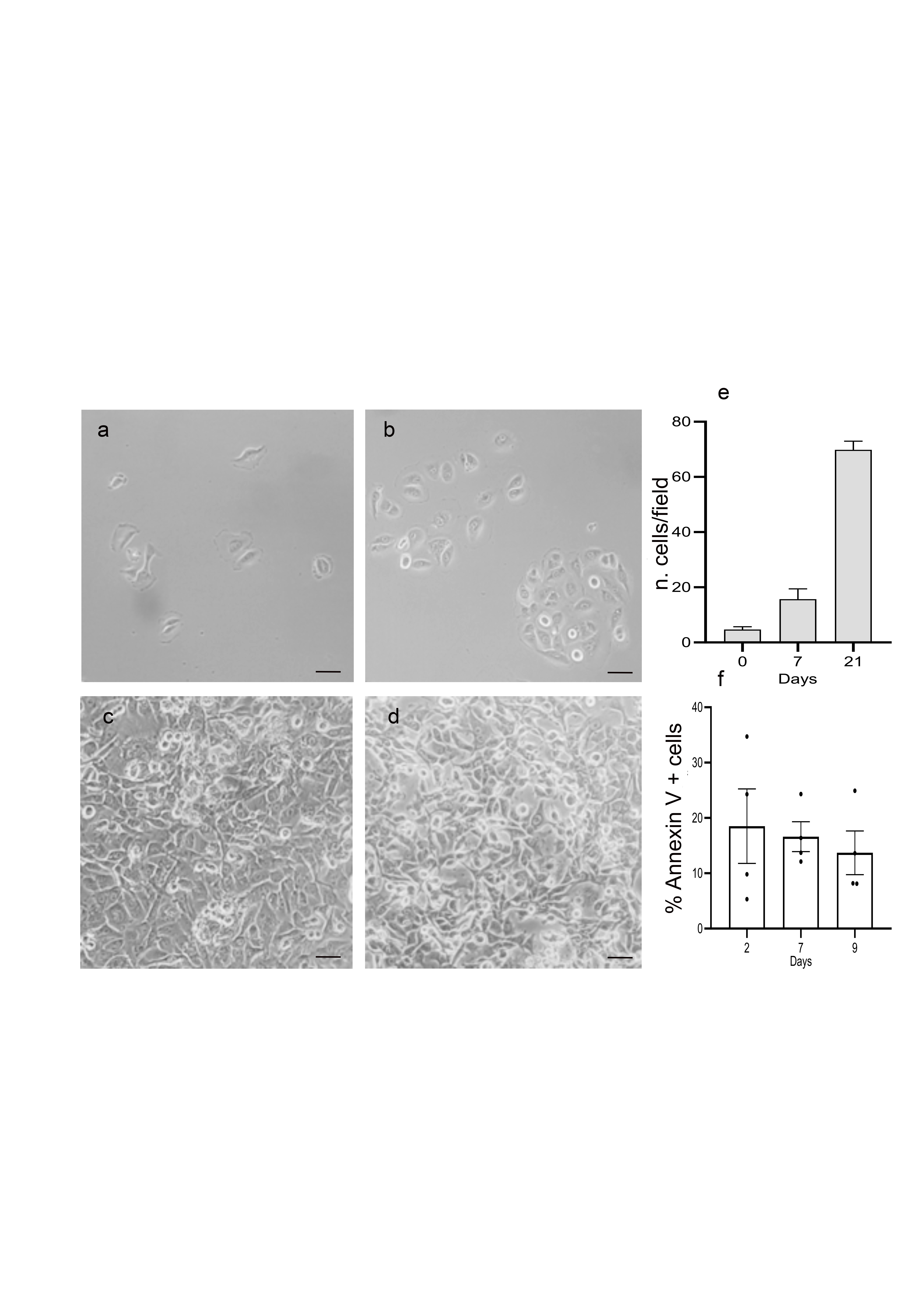

Supplement: Supplementary file 2 [file Image_1.jpg]

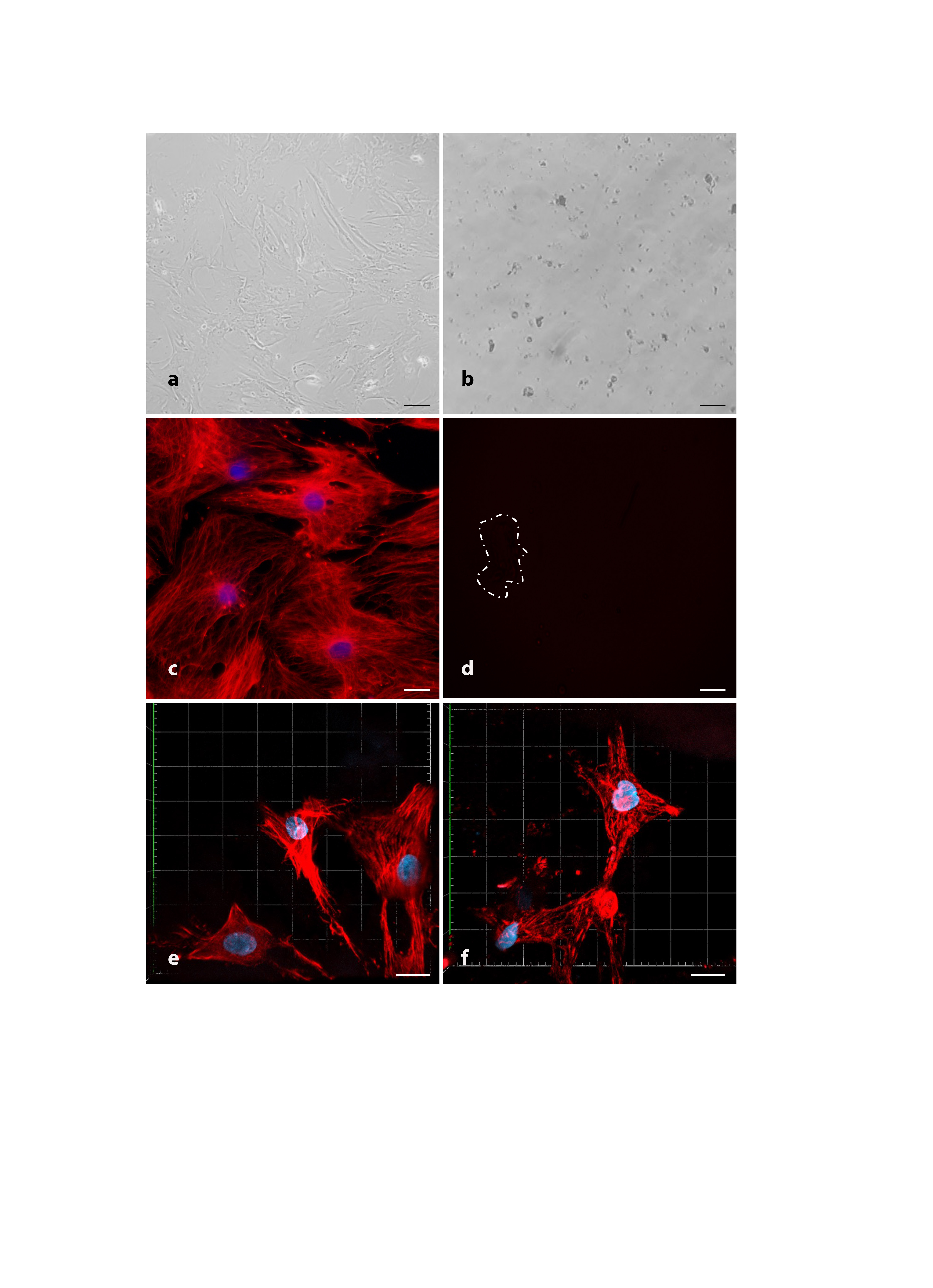

Supplement: Supplementary file 3 [file Image_2.jpg]

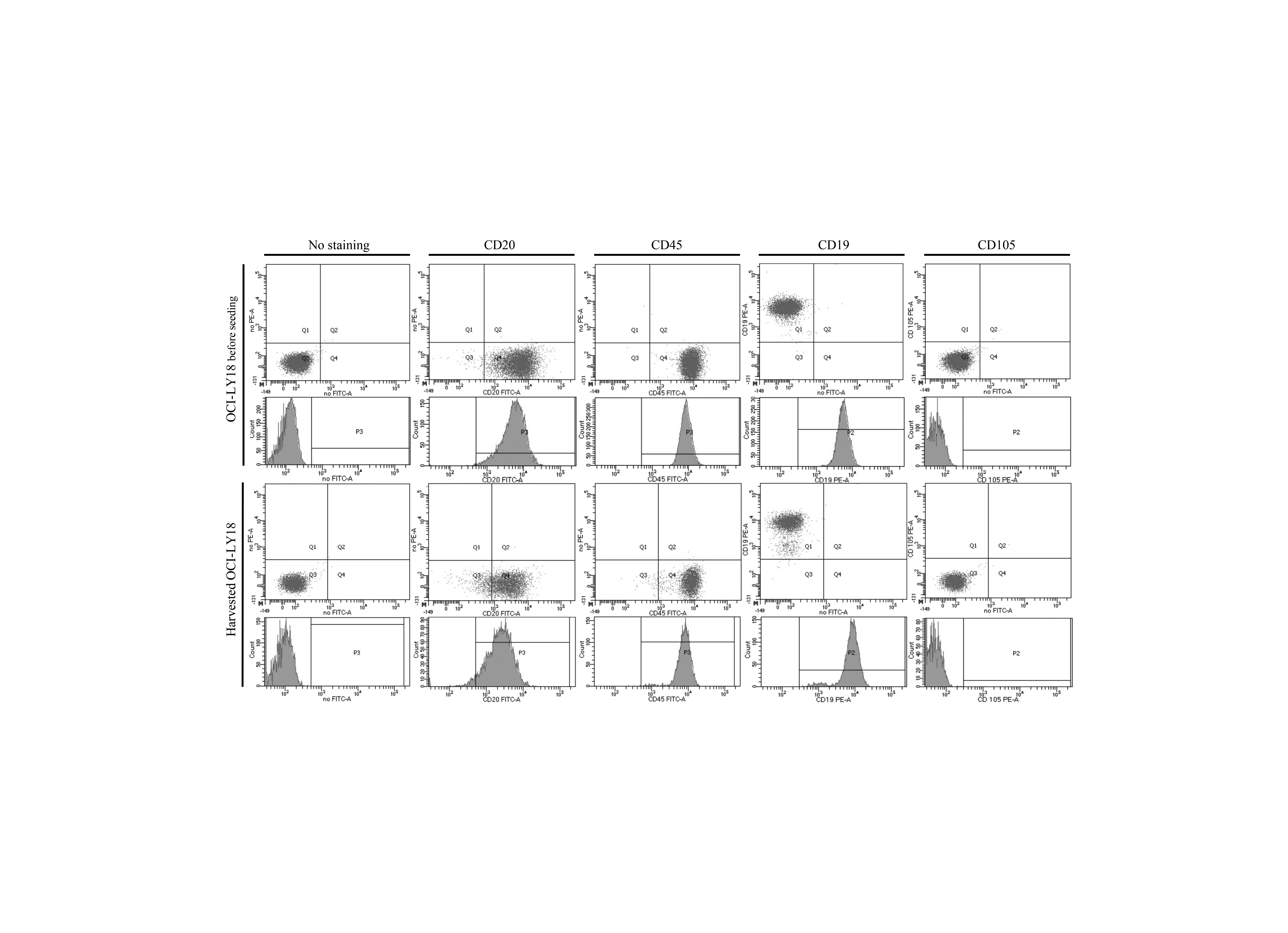

Supplement: Supplementary file 4 [file Image_3.jpg]

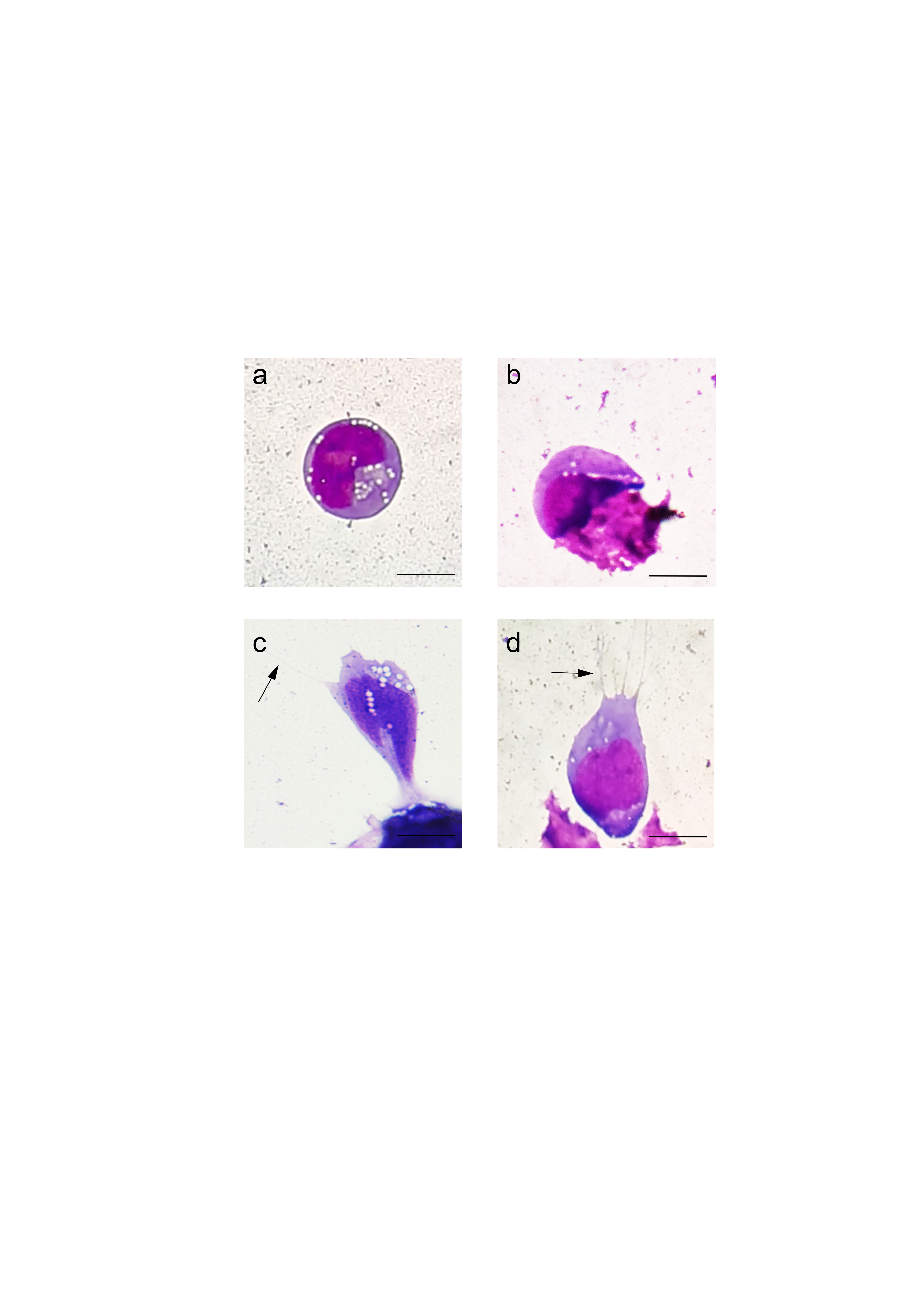

Supplement: Supplementary file 5 [file Image_4.jpg]
